# Challenges in Understanding Human-Algorithm Entanglement During Online Information Consumption

**DOI:** 10.1177/17456916231180809

**Published:** 2023-07-10

**Authors:** Stephan Lewandowsky, Ronald E. Robertson, Renee DiResta

**Affiliations:** 1 School of Psychological Science, University of Bristol; 2 Department of Psychology, University of Potsdam; 3 School of Psychological Science, University of Western Australia; 4 Stanford Internet Observatory, Stanford University

**Keywords:** algorithms, cognition, social cognition, social media

## Abstract

Most content consumed online is curated by proprietary algorithms deployed by social media platforms and search engines. In this article, we explore the interplay between these algorithms and human agency. Specifically, we consider the extent of entanglement or coupling between humans and algorithms along a continuum from implicit to explicit demand. We emphasize that the interactions people have with algorithms not only shape users’ experiences in that moment but because of the mutually shaping nature of such systems can also have longer-term effects through modifications of the underlying social-network structure. Understanding these mutually shaping systems is challenging given that researchers presently lack access to relevant platform data. We argue that increased transparency, more data sharing, and greater protections for external researchers examining the algorithms are required to help researchers better understand the entanglement between humans and algorithms. This better understanding is essential to support the development of algorithms with greater benefits and fewer risks to the public.

Much of the information people encounter online is curated by proprietary algorithms deployed by the platforms people interact with. Technically, an “algorithm” is simply “a series of steps undertaken in order to solve a particular problem or accomplish a defined outcome” (Diakopoulos, 2015, p. 400). However, the term is colloquially used to refer to the opaque systems that determine what users see online when using social media or web search engines. In such contexts, the term typically refers to an “algorithmic system,” which can be more broadly defined as “any socio-technical system influenced by at least one algorithm . . . [including] systems that may rely on human judgement and/or other non-algorithmic components, as long as they include at least one algorithm” (Bandy, 2021, p. 744). Understanding the interaction between human users and online algorithms thus presents an intricate psychological problem that requires an in-depth understanding of human cognition as well as technology.

Given the scarcity of human attention (Simon, 1971), algorithms that filter and curate content are essential to harness the abundance of information on the web: Googling “Newcastle” should return different results in Australia than in the United Kingdom, and without such filtering, much useful information would remain inaccessible. Likewise, recommender systems can help people satisfy their preferences, such as when they help people find movies, books, or restaurants that they are likely to enjoy (Ricci et al., 2015). Unsurprisingly, the public is mainly appreciative of algorithmic customization in such consumer contexts (Kozyreva et al., 2021).

Although algorithms across the web have delivered many benefits to users, they also—and perhaps mainly—deliver benefits to the platforms, which rely on algorithms to commodify human attention (Wu, 2017). Platforms primarily make money by displaying ads to users, and they can display more ads if users spend more time on site—hence, ultimately, most algorithms seek to increase time spent on the platform. This foundational incentive may explain why researchers have identified a number of adverse consequences of algorithms. Although those consequences may affect many aspects of life, here we focus mainly on psychological and societal fallouts within political and broader societal communication.

First, satisfying user preferences is not unconditionally good for either the user or society at large. For example, problems arise when algorithms suggest radicalizing or extremist material (Kaiser & Rauchfleisch, 2020) or when messages are microtargeted at people on the basis of their particular vulnerabilities (for a discussion, see Lorenz-Spreen et al., 2021). Second, the design and operation of such algorithms are proprietary and not readily subject to public scrutiny. Neither individual users nor society at large know in detail why search results or social media feeds are curated in a particular way (Pasquale, 2015).

Indeed, the public may not even realize that algorithms are at work at all when using social media (Eslami et al., 2015; Powers, 2017; Rader & Gray, 2015). Third, to complicate matters further, algorithms not only shape user behavior, but also, user responses in turn shape algorithmic outputs, contributing to “feedback loops” (Mansoury et al., 2020; Sun et al., 2019) and a “mutually-shaping system” (Boczkowski, 1999) that is difficult to understand and analyze.

In this article, we consider the extent of entanglement between humans and algorithms by placing popular algorithms along a continuum from implicit to explicit user demands. (We use “demand” to refer to the user’s preferences, and we use “curation” to describe algorithms’ ability to infer or use those demands to curate the content shown to users.) To do so, we examine the extent to which various algorithmic features generate their outputs on the basis of users’ explicitly provided preferences (e.g., channel subscriptions on YouTube) or implicitly provided preferences (e.g., dwell time on Facebook). While examining cases that fit along this continuum, we note that the interactions people have with algorithms not only shape what they are exposed to in that moment but, because of feedback loops (Mansoury et al., 2020; Sun et al., 2019) and the mutually shaping nature of such systems (Aral, 2020), can also have long-term or broader network effects. Collaborative filtering techniques in recommender systems, for example, draw on signal from some users to predict the preferences and shape the suggestions displayed to other users that the system perceives as similar. Once a user acts on those recommendations (e.g., by purchasing one of the books suggested by Amazon in its cross-promotional listings), the similarity matrix between that user and others is updated, which in turn changes the recommendations offered by the system in the future.

## The Entanglement of Humans and Algorithms

There is considerable diversity in the algorithms used by different platforms. For example, when people talk about Facebook’s algorithm, they tend to refer to the system curating their NewsFeed, whereas mentions of Google’s algorithm refer to the system curating web search results, and mentions of TikTok’s algorithm typically refer to the system that curates their “For You Page.” In addition, the interfaces used by online platforms vary widely in form (e.g., a ranked list vs. a stack) and function (e.g., active or passive information search) and can involve the presence of multiple algorithmic features (e.g., Facebook’s “People You May Know” feature; Zignani et al., 2014) that are continuously being updated through large-scale experimentation (Kramer et al., 2014). Given these differences and recent research highlighting the role of user choice in online platforms, we examine human-algorithm entanglement by placing specific algorithmic features on a continuum based on the extent to which their output is based on implicit or explicit user preferences.

Implicit preferences include proxies for attention that users may be unaware of (e.g., dwell time and mouse hovering) and those inferred by the platform (e.g., recommendations obtained through collaborative filtering). This implicit end of the implicit-explicit continuum includes algorithmic features that involve little or no active user input, such as Twitter’s #Explore “Trending” feature, which curates posts from across the platform writ large for topics or phrases it classifies as “trending.” Less extreme—but still largely driven by implicit preferences—are algorithmic features that are designed for content discovery, such as TikTok’s “For You Page,” YouTube’s “Stories,” or Instagram’s “Reels,” which rely heavily on collaborative filtering.

In contrast, explicit user preferences include both direct user inputs (e.g., clicks, queries, and follows/subscriptions) and inferred preferences based on those inputs (e.g., content-based filtering, which recommends content to users based on their own prior actions and engagement). For example, Instagram’s “Following” and “Favorites” features allow users to view chronologically arranged content from the accounts that they explicitly expressed a preference for. Perhaps the most explicit algorithms are those driving search engines. Unlike the feeds or recommendations on social media, which provide a more passive form of content curation, obtaining a page of search results requires that a user first composes and enters a search query.

Most algorithmic features are, however, located away from the endpoints of the implicit-explicit continuum because they incorporate a blend of implicit and explicit user preferences. To illustrate, Facebook’s Newsfeed and Twitter’s “For You Page” include not only content from the accounts that a user has explicitly expressed a preference for by following but also content that the platform selects to satisfy a user’s implicitly expressed presumed preferences. The capacity to differentiate which of an algorithm’s actions are due to implicit or explicit user preferences can be crucial to “algorithm audits” (Metaxa et al., 2021) that aim to understand why specific pieces of content are shown to certain individuals or in response to certain inputs. This differentiation may, however, be difficult to achieve. A case in point are YouTube’s video recommendations, which are based on both implicit and explicit user preferences. On one hand, a recent study found that explicit preferences (e.g., subscriptions) can best explain why some individuals are recommended more extremist videos on YouTube (Chen et al., 2022). On the other hand, a recent systematic review of the literature on the effects of YouTube’s recommender system on radicalization found that 14 out of 23 studies implicated the recommender system in facilitating access to problematic content compared with only two that argued the system was not involved (Yesilada & Lewandowsky, 2022).

The results returned by search engines are a particularly interesting case of subtle entanglement between humans and algorithms. At the implicit level, search engines adjust results based on a person’s implicit preferences that have been recorded or inferred by the platform. For example, localization, the automated tailoring of results to a user’s inferred geolocation, has been shown to be a powerful driver of differences between individuals’ search results (Kliman-Silver et al., 2015). By contrast, the extent of personalization—defined as content curation tailored to an individual (Beam, 2014; Sundar, 2020)—is low on Google Search (Robertson et al., 2018). At the explicit level, the most powerful driver of differences between individuals’ search results is the composition of the user’s search query itself (Robertson et al., 2018). Though there is to some degree a mainstreaming effect, in which similar queries produce similar search results (Trielli & Diakopoulos, 2022), people tend to formulate queries that contain subtle partisan signals reflecting their ideology (Mustafaraj et al., 2020; Trielli & Diakopoulos, 2022; van Hoof et al., 2022). For example, an analysis of political search terms during the 2018 midterm elections revealed that conservatives are more likely to search for the background of candidates (e.g., “sherrod brown background,” “party jim rennaci”), whereas liberals are more likely to search for candidates’ positions on issues (e.g., “beto stand issues,” “beto policy weed”; Trielli & Diakopoulos, 2022).

Politically related search results therefore appear to be primarily determined by users’ own explicit demands, as reflected by their queries and selection behavior (Robertson et al., 2023), although research on other important issues in web search implicates the algorithms and underlying data they use (Noble, 2018; Vlasceanu & Amodio, 2022).

These intermediate cases on the implicit–explicit continuum help illustrate how identifying the role of implicit and explicit user preferences in shaping an algorithm’s outputs can be a complex but valuable task. The complexity of this task can also be influenced by a wide range of additional factors worth considering, including (a) the time horizons that algorithms operate on; (b) the dynamics between users, content creators, and platforms; and (c) the mutually shaping nature of these systems. With respect to the time horizons, an explicit signal on one day may continue to inform the decisions of a recommender system for an indeterminate amount of time, such as when users continue to see an ad for an item they have already purchased.

Understanding the role of implicit and explicit demand in human-algorithm entanglement requires examination not just of user behavior but also the behavior of the people who create content and the platforms that host it. Users vary in terms of their algorithmic awareness or literacy, and they also differ in the extent to which they use that presumed knowledge in further attempts to exert control over an algorithm’s outputs. For example, users may develop folk theories about how an algorithm works, which may alter their behavior as they attempt to gain greater control (Martens et al., 2023). Likewise, content creators who strive to be “algorithmically recognizable” (Gillespie, 2017) or who seek to game algorithms to amplify unreliable (Bradshaw, 2019) or fake (Elmas et al., 2021) content can also affect algorithmic outputs and in turn user behavior. Gaps in search coverage (known as “data voids”) can be exploited by malicious actors (Golebiewski & boyd, 2019). To illustrate, few people ever searched for “Sutherland Springs,” a small town in Texas, before a mass shooting occurred on November 4, 2017. Because there was little online content about Sutherland Springs at the time (barring weather information, a map, and a Wikipedia entry), malicious actors were able to influence search rankings by posting a torrent of material that (falsely) blamed the shooting on the “Antifa” movement. These malicious actors succeeded in shaping the front page of search queries and even injected “Antifa” into auto-suggest. There is evidence that this was no isolated incident and that white supremacists systematically seek to exploit data voids that can be filled with extremist material against little competition (Golebiewski & boyd, 2019).

Human-algorithm entanglement also appears in algorithmic content moderation, such as via account downranking (sometimes called “shadow banning” by users and “soft actions” by platforms), which gives the platforms a way to reduce the reach of an account without actually removing it. However, such opaque decisions can create controversy because they intervene on users’ explicit preferences (by not showing them content from accounts they follow) in ways that are hard to identify without access to internal data.

The mutually shaping nature of human-algorithmic systems also requires consideration of the broader context and ecosystem that they operate in (Zuckerman, 2021). That is, irrespective of the degree of their entanglement with humans, algorithms can also contribute to the shaping of the various networks—whether social networks (in the case of social media) or the web more broadly (in the case of search engines)—that underlie their outputs. Some algorithms function for that exact purpose—growing or altering their underlying information networks, also often referred to as “substrate” (Aral, 2020). By making suggestions about who to connect to via “people recommender systems” (Fabbri et al., 2022)—for example, providing information about friends of friends on Facebook or suggesting who to follow on Twitter—such algorithms can connect users to growing communities on the platform that align with their interests and preferences. Those lasting downstream consequences of algorithms make it even more urgent that a better understanding of their inner workings is gained.

## The Opacity of Algorithmic Systems

Despite their central role in directing people to online information and in contributing to the construction of the substrate, relatively little is known about the algorithms used by major platforms and their psychological and societal impacts (Jarsulic, 2022; Rahwan et al., 2019; Wagner et al., 2021). There are several reasons for this paucity of knowledge. First and foremost, visibility into the workings and impact of the algorithms is limited by lack of access to relevant data, such as user input and actions and algorithm outputs.

At times, access to the data required to evaluate an algorithm is actively curtailed by the platforms, which have been known to engage in vigorous efforts to shut down independent academic research, including via legal means (Greene et al., 2022; this despite the fact that transparency has been shown to frequently be in a firm’s interest, Wang et al., 2023). As a result, when concerning information has become available, it often involved whistleblowers. For example, in 2021, a former Facebook employee, Frances Haugen, revealed that Facebook’s newsfeed curation algorithm favored material that made people emotional (including sad and angry) over material that elicited a “like” by a factor of 5 (Kallioniemi, 2022). Anger-evoking material is disproportionately likely to include misinformation, toxicity, hate speech, and low-quality news (Paschen, 2019). It also became known that Facebook’s recommendation engines were pushing people into extreme groups and conspiracy theories, such as QAnon (Zadrozny, 2021). Although aware of those problems, Facebook chose not to take any action and thus “systematically amped up some of the worst of its platform, making it more prominent in users’ feeds and spreading it to a much wider audience” (Merrill & Oremus, 2021).

Nonetheless, focus is restricted to the observable interactions between users and algorithms on a single platform, which can be described in terms of what users were shown (exposure) and what users did (engagement; Robertson et al., 2023), research can still provide a useful “basis for understanding global cross-platform flows” (Zuckerman, 2021, p. 1509). Within this narrowed context, work from both platform-internal and independent external researchers can help provide a foundation for more broadly understanding how algorithms affect users.

### Internal research

Studies conducted internally by social media platforms benefit from access to full exposure and engagement data—what real users were shown and what they selected during their everyday use of a platform. In other words, internal research has access to users’ implicit demand and how that interacts with algorithmic interventions. To illustrate, researchers at Facebook have published experiments on their newsfeed algorithms that aimed to mobilize voters (Bond et al., 2012), tested for emotional contagion (Kramer et al., 2014), and measured selective exposure to ideologically congruent content (Bakshy et al., 2015). This research has not been without controversy. For example, Kramer et al. (2014) involved nearly 700,000 Facebook users in their experiment without people’s explicit consent (consent was taken to be implied because Facebook’s terms and conditions include reference to research). The experiment interfered with standard algorithmic curation by reducing the amount of emotional content in the newsfeed. When expression of positive emotion was reduced, users produced fewer positive and more negative posts, and the converse was observed when negative emotion was reduced.

Likewise, researchers from Twitter recently published the results of a long-term experiment that altered the default newsfeed algorithm to examine how it amplifies political information (Huszár et al., 2022). Most recently, LinkedIn collaborated with external researchers to investigate the impact of their “People You May Know” algorithm with respect to the “strength of weak ties” hypothesis (Rajkumar et al., 2022), a long-standing sociological theory that posits the value of weak social ties in obtaining a job (Granovetter, 1973).

Although these internal research efforts provide useful insight into each platform’s algorithms, they carry their own risks. One risk is that if the research results are considered controversial or problematic—as occurred with the research on emotional contagion (Kramer et al., 2014)—the ensuing public backlash may keep further platform-sponsored research from being published. Another risk is the obvious lack of independence, which may also lead to research not being published even though the findings might be in the public interest. For that reason, independent external research is particularly crucial.

### External research

External researchers have explored several avenues to overcome the lack of data availability for auditing algorithms. This includes using a platform’s official API (application programming interface) to obtain the data that is made accessible, collecting data through simulated user-algorithm interactions (Kawakami et al., 2020), or seeking volunteers among users of the platforms who consent to share their personal data with researchers and are given assurances that their privacy will be preserved. The latter approach has repeatedly been thwarted by Facebook, often entailing threats of legal action (Brandom, 2021). Common to all those approaches is the idea that algorithms can be reverse engineered by seeking to infer an algorithm’s design on the basis of its observable behavior (Bandy, 2021; Diakopoulos, 2015; Metaxa et al., 2021). Reverse engineering can range from the relatively simple (e.g., examining which words are excluded from autocorrect on the iPhone; Keller, 2013) to the highly complex (e.g., an analysis of how political ads are delivered on Facebook; Ali et al., 2019).

The reverse engineering through external methods has uncovered several problematic aspects of algorithms, such as discriminatory advertising practices and stereotypical representations of Black Americans in Google Search (Noble, 2018; Sweeney, 2013) and in the autocomplete suggestions that Google provides when entering search terms (Baker & Potts, 2013). In other cases, such investigations have helped shed light on interactions between users and the algorithms and in some recent cases, have pointed to the importance of users’ choices in explaining the algorithmically curated content they are exposed to (Chen et al., 2022; Robertson et al., 2023). These results are, however, limited by the fact that researchers can monitor behavior for only so long and do not have access to historical data, which in turn restricts focus mainly to exploring users’ explicit demands. In consequence, it is impossible to rule out that the small number of people identified in these studies who disproportionately consume the vast majority of problematic content were previously pushed into that state by the platform algorithms. Likewise, current audits of the YouTube recommender algorithm cannot investigate recommendations made before YouTube took action to clean up the algorithm and to de-emphasize conspiratorial content (YouTube, n.d.).

## The Missing Link: Transparency

Most research to date has involved one-off studies that tell something about how an algorithm operates under a specific set of conditions, on a specific platform, and at the time the study was conducted. Often, those studies lose relevance and validity when the world changes (Munger, 2019), which can happen in an instant at the whim of a platform’s decision to alter its policies or algorithms (Reuning et al., 2022). The takeover of Twitter by Elon Musk in late 2022 and the ensuing erratic policy decisions have brought this risk into sharp focus.

This situation can be redressed only by enhancing researcher access to data around the signals that ultimately drive the algorithms—that is, users’ personal data, behavior, and exposure to posts—and that are currently being closely guarded by the platforms (Suzor et al., 2019).

Recent voluntary transparency measures by the platforms (e.g., Facebook’s “ad library”) are insufficient to analyze political microtargeting and to fully understand what content has been shown (Dommett & Power, 2019). The ad library is also missing more than 100,000 political ads (Edelson & McCoy, 2021).

The need for data access by researchers has been recognized politically, with the recently enacted Digital Services Act (DSA) in the European Union and the Platform Accountability and Transparency Act (PATA) recently proposed by U.S. Senators Christopher A. Coons (D-Del), Rob Portman (R-Ohio), and Amy Klobuchar (D-Minn; Nonnecke & Carlton, 2022). PATA would require social media companies to provide researchers vetted by a third-party body, such as the National Science Foundation, access to certain platform data in response to approved research requests. The DSA similarly mandates that platforms cooperate with independent audits of compliance with their obligations, including audits of their algorithms. The DSA specifically includes civil-society organizations and other research organizations among the institutions that may gather access to platform data for research and auditing purposes. Crucially, the commercial interests of platforms are not sufficient grounds to refuse data access for research and auditing purposes. To support the enforcement of the DSA, the European Commission’s Joint Research Centre is establishing the European Centre for Algorithmic Transparency (ECAT), which will support Europe’s efforts in regulating digital services and in particular, the algorithmic systems that power them. In addition, ECAT will serve as an international research hub with a focus on algorithmic systems, algorithmic transparency, and artificial intelligence (ECAT, n.d.). The prominence of requiring data access by researchers in both legislative initiatives is indicative of the recognized importance of platform transparency.

There are, however, also dark sides to transparency. For example, when platforms are exhaustively transparent about what the rules are, that transparency can be used by bad-faith actors to “ride the line” without technically crossing it, thereby flooding the platform with problematic content that cannot be subject to moderation or removal. To illustrate, anti-vaccination influencers on Instagram have been shown to develop strategies to circumvent content moderation based on elaborate “folk” theories of visibility on the platform (Moran et al., 2022). Arguably, this capability would be enhanced by greater transparency.

Likewise, platforms such as Facebook may be unable to be fully transparent about what is being used to target consumers with advertisements. While Facebook has, of course, knowledge of the interests of the users that an advertiser has selected for targeting, the platform has no access to advertisers’ true intentions. If advertisers know how to infer a latent variable, such as personality, from interests or likes (Youyou et al., 2015) and they target users based on personality rather than the interests that are merely proxy variables, then Facebook cannot be transparent about that without creating a battery of models that can reverse engineer advertisers’ true intentions from their selection of surface feature. It is unclear how platforms or regulators should respond. Maybe the platforms should develop such tools to infer advertisers’ true intent.

Alternatively, maybe there should be a limit to the granularity of targeting so that the harm is limited even if it is not known what the harm could be. It is known, however, that the public overwhelmingly rejects manipulative politically motivated targeting (Kozyreva et al., 2021), which operates as a form of invisible influence, with its operations hidden to the user (Susser et al., 2019).

Reconciling the need for transparency with threats to privacy and exploitation and finding that sweet spot where algorithms are helpful but not manipulative will require many complex and nuanced conversations between platforms, users, policymakers, and the public at large. Lewandowsky and Pomerantsev (2022) provided a brief sketch of what such an “Internet with democratic credentials” might look like. In Europe, ECAT could potentially exercise its convening power to move the agenda forward with an inclusive approach to stakeholder involvement, although at this stage, it remains unclear to what extent ECAT’s research agenda will tackle a whole-of-society approach.

## Conclusions

Words and actions online do not stay online. The violent storming of the U.S. Capitol on January 6, 2021, was organized online and starkly illustrates the real-world impact of toxic online rhetoric (Frenkel, 2021). The possibility that algorithms amplify such toxic voices online must therefore give rise to particular concern. Facebook has been causally implicated in violent hate crimes against refugees in Germany (Müller & Schwarz, 2021), and there is now considerable evidence that Facebook use causes political polarization (Lauer, 2021).

Simply turning off algorithms is not a solution. Some voices have called for a replacement of newsfeed algorithms by a strictly temporal (nonalgorithmic) feed that simply shows posts as they come in, one by one. However, this solution is itself an algorithm, called “exclusively recency-based,” that introduces its own bias. For example, strict temporal presentation favors super-posters (e.g., paid “trolls”) because the more people post, the more likely they are to contribute the most recent event that users consume. This is a bias that does not qualitatively differ from the biases of other algorithms. People will therefore remain entangled with algorithms online, and researchers’ goal should be to seek greater understanding and better management of that entanglement.

## Transparency

*Action Editor:* Melanie Mitchell

*Editor:* Interim Editorial Panel

## References

[bibr1-17456916231180809] Ali M. Sapiezynski P. Korolova A. Mislove A. Rieke A. (2019). Ad delivery algorithms: The hidden arbiters of political messaging. arXiv. 10.48550/arXiv.1912.04255

[bibr2-17456916231180809] Aral S. (2020). The hype machine: How social media disrupts our elections, our economy, and our health—and how we must adapt. Penguin Random House.

[bibr3-17456916231180809] Baker P. Potts A. (2013). ‘Why do white people have thin lips?’ Google and the perpetuation of stereotypes via auto-complete search forms. Critical Discourse Studies, 10, 187–204. 10.1080/17405904.2012.744320

[bibr4-17456916231180809] Bakshy E. Messing S. Adamic L. A. (2015). Exposure to ideologically diverse news and opinion on Facebook. Science, 348(6239), 1130–1132. 10.1126/science.aaa1160 25953820

[bibr5-17456916231180809] Bandy J. (2021). Problematic machine behavior: A systematic literature review of algorithm audits. Proceedings of the ACM on Human-Computer Interaction, 5(CSCW1), 74:1–74:34. 10.1145/344914836644216

[bibr6-17456916231180809] Beam M. A. (2014). Automating the news: How personalized news recommender system design choices impact news reception. Communication Research, 41(8), 1019–1041. 10.1177/0093650213497979

[bibr7-17456916231180809] Boczkowski P. (1999). Mutual shaping of users and technologies in a national virtual community. Journal of Communication, 49, 86–108. 10.1111/j.1460-2466.1999.tb02795.x

[bibr8-17456916231180809] Bond R. M. Fariss C. J. Jones J. J. Kramer A. D. I. Marlow C. Settle J. E. Fowler J. H. (2012). A 61-million-person experiment in social influence and political mobilization. Nature, 489(7415), 295–298. 10.1038/nature11421 22972300 PMC3834737

[bibr9-17456916231180809] Bradshaw S. (2019). Disinformation optimised: Gaming search engine algorithms to amplify junk news. Internet Policy Review, 8(4). 10.14763/2019.4.1442

[bibr10-17456916231180809] Brandom R. (2021, August). Facebook shut down German research on Instagram algorithm, researchers say. The Verge. https://www.theverge.com/2021/8/13/22623354/facebook-instagram-algorithm-watch-research-legal-threat

[bibr11-17456916231180809] Chen A. Y. Nyhan B. Reifler J. Robertson R. E. Wilson C. (2022). Subscriptions and external links help drive resentful users to alternative and extremist YouTube. arXiv 10.48550/arXiv.2204.10921 PMC10468121 37647396

[bibr12-17456916231180809] Diakopoulos N. (2015). Algorithmic accountability. Digital Journalism, 3, 398–415. 10.1080/21670811.2014.976411

[bibr13-17456916231180809] Dommett K. Power S. (2019). The political economy of Facebook advertising: Election spending, regulation and targeting online. The Political Quarterly, 90(2), 257–265. 10.1111/1467-923x.12687

[bibr14-17456916231180809] Edelson L. McCoy D. (2021, August 10). We research misinformation on Facebook. It just disabled our accounts. The New York Times. https://www.nytimes.com/2021/08/10/opinion/facebook-misinformation.html

[bibr15-17456916231180809] Elmas T. Overdorf R. Özkalay A. F. Aberer K. (2021). Ephemeral astroturfing attacks: The case of fake Twitter trends. In 2021 IEEE European symposium on security and privacy (EuroS&P) (pp. 403–422). IEEE. 10.1109/EuroSP51992.2021.00035

[bibr16-17456916231180809] Eslami M. Rickman A. Vaccaro K. Aleyasen A. Vuong A. Karahalios K. Hamilton K. Sandvig C. (2015). “I always assumed that I wasn’t really that close to [her]” reasoning about invisible algorithms in news feeds. In Proceedings of the 33rd annual ACM conference on human factors in computing systems (pp. 153–162). Association for Computing Machinery.

[bibr17-17456916231180809] European Centre for Algorithmic Transparency. (n.d.). [Home page]. https://algorithmic-transparency.ec.europa.eu/index_en

[bibr18-17456916231180809] Fabbri F. Croci M. L. Bonchi F. Castillo C. (2022). Exposure inequality in people recommender systems: The long-term effects. Proceedings of the International AAAI Conference on Web and Social Media, 16, 194–204. https://ojs.aaai.org/index.php/ICWSM/article/view/19284

[bibr19-17456916231180809] Frenkel S. (2021, January 6). The storming of Capitol Hill was organized on social media. The New York Times. https://www.nytimes.com/2021/01/06/us/politics/protesters-storm-capitol-hill-building.html

[bibr20-17456916231180809] Gillespie T. (2017). Algorithmically recognizable: Santorum’s Google problem, and Google’s Santorum problem. Information, Communication & Society, 20(1), 63–80. 10.1080/1369118X.2016.1199721

[bibr21-17456916231180809] Golebiewski M. boyd d. (2019). Data voids: Where missing data can easily be exploited. Data & Society.

[bibr22-17456916231180809] Granovetter M. S. (1973). The strength of weak ties. American Journal of Sociology, 78(6), 1360–1380. https://www.jstor.org/stable/2776392

[bibr23-17456916231180809] Greene T. Martens D. Shmueli G. (2022). Barriers to academic data science research in the new realm of algorithmic behaviour modification by digital platforms. Nature Machine Intelligence, 4, 323–330. 10.1038/s42256-022-00475-7

[bibr24-17456916231180809] Huszár F. Ktena S. I. O’Brien C. Belli L. Schlaikjer A. Hardt M. (2022). Algorithmic amplification of politics on Twitter. Proceedings of the National Academy of Sciences, USA, 119(1), Article e2025334119. 10.1073/pnas.2025334119 PMC8740571 34934011

[bibr25-17456916231180809] Jarsulic M. (2022). Addressing the competitive harms of opaque online surveillance and recommendation algorithms. The Antitrust Bulletin, 67, 100–112. 10.1177/0003603X211066983

[bibr26-17456916231180809] Kaiser J. Rauchfleisch A. (2020). Birds of a feather get recommended together: Algorithmic homophily in YouTube’s channel recommendations in the United States and Germany. Social Media + Society, 6(4). 10.1177/2056305120969914

[bibr27-17456916231180809] Kallioniemi P. (2022). Facebook’s dark pattern design, public relations and internal work culture. Journal of Digital Media & Interaction, 5(12), 38–54. 10.34624/JDMI.V5I12.28378

[bibr28-17456916231180809] Kawakami A. Umarova K. Mustafaraj E. (2020). The media coverage of the 2020 US presidential election candidates through the lens of Google’s top stories. In Proceedings of the international AAAI conference on web and social media (Vol. 14, pp. 868–877). PKP Publishing Services Network. https://aaai.org/ojs/index.php/ICWSM/article/view/7352

[bibr29-17456916231180809] Keller M. (2013, July). The Apple ‘kill list’: What your iPhone doesn’t want you to type. Daily Beast. https://www.thedailybeast.com/the-apple-kill-list-what-your-iphone-doesnt-want-you-to-type

[bibr30-17456916231180809] Kliman-Silver C. Hannak A. Lazer D. Wilson C. Mislove A. (2015). Location, location, location: The impact of geolocation on web search personalization. In Proceedings of the 2015 ACM conference on internet measurement conference - IMC ’15 (pp. 121–127). ACM Press. 10.1145/2815675.2815714

[bibr31-17456916231180809] Kozyreva A. Lorenz-Spreen P. Hertwig R. Lewandowsky S. Herzog S. M. (2021). Public attitudes towards algorithmic personalization and use of personal data online: Evidence from Germany, Great Britain, and the United States. Humanities and Social Sciences Communications, 8, Article 117. 10.1057/s41599-021-00787-w

[bibr32-17456916231180809] Kramer A. D. I. Guillory J. E. Hancock J. T. (2014). Experimental evidence of massive-scale emotional contagion through social networks. Proceedings of the National Academy of Sciences, USA, 111(24), 8788–8790. 10.1073/pnas.1320040111 PMC4066473 24889601

[bibr33-17456916231180809] Lauer D. (2021). Facebook’s ethical failures are not accidental; They are part of the business model. AI and Ethics, 1, 395–403. 10.1007/s43681-021-00068-x 34790950 PMC8179701

[bibr34-17456916231180809] Lewandowsky S. Pomerantsev P. (2022). Technology and democracy: A paradox wrapped in a contradiction inside an irony. Memory, Mind & Media, 1, Article E5. 10.1017/mem.2021.7 PMC7613775 36415623

[bibr35-17456916231180809] Lorenz-Spreen P. Geers M. Pachur T. Hertwig R. Lewandowsky S. Herzog S. M. (2021). Boosting people’s ability to detect microtargeted advertising. Scientific Reports, 11, Article 15541. 10.1038/s41598-021-94796-z PMC8324838 34330948

[bibr36-17456916231180809] Mansoury M. Abdollahpouri H. Pechenizkiy M. Mobasher B. Burke R. (2020). Feedback loop and bias amplification in recommender systems. In Proceedings of the 29th ACM international conference on information & knowledge management. Association for Computing Machinery.

[bibr37-17456916231180809] Martens M. De Wolf R. Berendt B. De Marez L. (2023). Decoding algorithms: Exploring end-users’ mental models of the inner workings of algorithmic news recommenders. Digital Journalism, 11, 203–225. 10.1080/21670811.2022.2129402

[bibr38-17456916231180809] Merrill J. B. Oremus W. (2021, October 26). Five points for anger, one for a ‘like’: How Facebook’s formula fostered rage and misinformation. The Washington Post. https://www.washingtonpost.com/technology/2021/10/26/facebook-angry-emoji-algorithm/

[bibr39-17456916231180809] Metaxa D. Park J. S. Robertson R. E. Karahalios K. Wilson C. Hancock J. Sandvig C. (2021). Auditing algorithms: Understanding algorithmic systems from the outside in. Foundations and Trends® in Human–Computer Interaction, 14(4), 272–344. 10.1561/1100000083

[bibr40-17456916231180809] Moran R. E. Grasso I. Koltai K. (2022). Folk theories of avoiding content moderation: How vaccine-opposed influencers amplify vaccine opposition on Instagram. Social Media + Society, 8(4). 10.1177/20563051221144252

[bibr41-17456916231180809] Müller K. Schwarz C. (2021). Fanning the flames of hate: Social media and hate crime. Journal of the European Economic Association, 19, 2131–2167. 10.1093/jeea/jvaa045

[bibr42-17456916231180809] Munger K. (2019). The limited value of non-replicable field experiments in contexts with low temporal validity. Social Media + Society, 5(3). 10.1177/2056305119859294

[bibr43-17456916231180809] Mustafaraj E. Lurie E. Devine C. (2020). The case for voter-centered audits of search engines during political elections. In FAT* ‘20: Proceedings of the 2020 Conference on Fairness, Accountability, and Transparency (pp. 559–569). Association for Computing Machinery.

[bibr44-17456916231180809] Noble S. U. (2018). Algorithms of oppression: How search engines reinforce racism. New York University Press. 10.1126/science.abm5861 34709921

[bibr45-17456916231180809] Nonnecke B. Carlton C. (2022). EU and US legislation seek to open up digital platform data. Science, 375, 610–612. 10.1126/science.abl8537 35143295

[bibr46-17456916231180809] Paschen J. (2019). Investigating the emotional appeal of fake news using artificial intelligence and human contributions. Journal of Product & Brand Management, 29, 223–233. 10.1108/jpbm-12-2018-2179

[bibr47-17456916231180809] Pasquale F. (2015). The black box society. Harvard University Press.

[bibr48-17456916231180809] Powers E. (2017). My news feed is filtered? Digital Journalism, 5, 1315–1335. 10.1080/21670811.2017.1286943

[bibr49-17456916231180809] Rader E. Gray R. (2015). Understanding user beliefs about algorithmic curation in the Facebook news feed. In Proceedings of the 33rd annual ACM conference on human factors in computing systems (pp. 173–182). Association for Computing Machinery. 10.1145/2702123.2702174

[bibr50-17456916231180809] Rahwan I. Cebrian M. Obradovich N. Bongard J. Bonnefon J.-F. Breazeal C. Crandall J. W. Christakis N. A. Couzin I. D. Jackson M. O. Jennings N. R. Kamar E. Kloumann I. M. Larochelle H. Lazer D. McElreath R. Mislove A. Parkes D. C. Pentland A. S. . . . Wellman M. (2019). Machine behaviour. Nature, 568(7753), 477–486. 10.1038/s41586-019-1138-y 31019318

[bibr51-17456916231180809] Rajkumar K. Saint-Jacques G. Bojinov I. Brynjolfsson E. Aral S. (2022). A causal test of the strength of weak ties. Science, 377(6612), 1304–1310. 10.1126/science.abl4476 36107999

[bibr52-17456916231180809] Reuning K. Whitesell A. Hannah A. L. (2022). Facebook algorithm changes may have amplified local republican parties. Research & Politics, 9(2). 10.1177/20531680221103809

[bibr53-17456916231180809] Ricci F. Rokach L. Shapira B. (2015). Recommender systems: Introduction and challenges. Springer.

[bibr54-17456916231180809] Robertson R. E. Green J. Ruck D. J. Ognyanova K. Wilson C. Lazer D. (2023). Users choose to engage with more partisan news than they are exposed to on Google Search. Nature, 618, 342–348. 10.1038/s41586-023-06078-5 37225979

[bibr55-17456916231180809] Robertson R. E. Jiang S. Joseph K. Friedland L. Lazer D. Wilson C. (2018). Auditing partisan audience bias within Google Search. Proceedings of the ACM on Human-Computer Interaction, 2, 1–22. 10.1145/3274417

[bibr56-17456916231180809] Simon H. A. (1971). Designing organizations for an information-rich world. In Greenberger M. (Ed.), Computers, communications and the public interest (Vol. 70, pp. 37–72). Johns Hopkins University Press.

[bibr57-17456916231180809] Sun W. Khenissi S. Nasraoui O. Shafto P. (2019). Debiasing the human-recommender system feedback loop in collaborative filtering. In Companion proceedings of the 2019 World Wide Web conference (pp. 645–651). Association for Computing Machinery. 10.1145/3308560.3317303

[bibr58-17456916231180809] Sundar S. S. (2020). Rise of machine agency: A framework for studying the psychology of human–AI interaction (HAII). Journal of Computer-Mediated Communication, 25(1), 74–88. 10.1093/jcmc/zmz026

[bibr59-17456916231180809] Susser D. Roessler B. Nissenbaum H. (2019). Online manipulation: Hidden influences in a digital world. Georgetown Law Technology Review, 4, 1–45.

[bibr60-17456916231180809] Suzor N. P. West S. M. Quodling A. York J. (2019). What do we mean when we talk about transparency? Toward meaningful transparency in commercial content moderation. International Journal of Communication, 13, 1526–1543.

[bibr61-17456916231180809] Sweeney L. (2013). Discrimination in online ad delivery. Queue, 11, 1–19. 10.1145/2460276.2460278

[bibr62-17456916231180809] Trielli D. Diakopoulos N. (2022). Partisan search behavior and Google results in the 2018 U.S. midterm elections. Information, Communication & Society, 25(1), 145–161. 10.1080/1369118X.2020.1764605

[bibr63-17456916231180809] van Hoof M. Meppelink C. S. Moeller J. Trilling D . (2022). Searching differently? How political attitudes impact search queries about political issues. New Media & Society. Advance online publication. 10.1177/14614448221104405

[bibr64-17456916231180809] Vlasceanu M. Amodio D. M. (2022). Propagation of societal gender inequality by internet search algorithms. Proceedings of the National Academy of Sciences, USA, 119(29), Article e2204529119. 10.1073/pnas.2204529119 PMC9304000 35858360

[bibr65-17456916231180809] Wagner C. Strohmaier M. Olteanu A. Kıcıman E. Contractor N. Eliassi-Rad T. (2021). Measuring algorithmically infused societies. Nature, 595(7866), 197–204. 10.1038/s41586-021-03666-1 34194046

[bibr66-17456916231180809] Wang Q. Huang Y. Jasin S. Singh P. V. (2023). Algorithmic transparency with strategic users. Management Science, 69(4), 1935–2545, iii–iv. 10.1287/mnsc.2022.4475

[bibr67-17456916231180809] Wu T. (2017). The attention merchants. Atlantic Books.

[bibr68-17456916231180809] Yesilada M. Lewandowsky S. (2022). Systematic review: YouTube recommendations and problematic content. Internet Policy Review, 11(1), Article 1652. 10.14763/2022.1.1652 PMC7613872 36466439

[bibr69-17456916231180809] YouTube. (n.d.). Community guidelines. https://www.youtube.com/howyoutubeworks/policies/community-guidelines/.

[bibr70-17456916231180809] Youyou W. Kosinski M. Stillwell D. (2015). Computer-based personality judgments are more accurate than those made by humans. Proceedings of the National Academy of Sciences, USA, 112, 1036–1040. 10.1073/pnas.1418680112 PMC4313801 25583507

[bibr71-17456916231180809] Zadrozny B. (2021, October). ‘Carol’s journey’: What Facebook knew about how it radicalized users. NBC News. https://www.nbcnews.com/tech/tech-news/facebook-knew-radicalized-users-rcna3581

[bibr72-17456916231180809] Zignani M. Gaito S. Rossi G. P. Zhao X. Zheng H. Zhao B. (2014). Link and triadic closure delay: Temporal metrics for social network dynamics. Proceedings of the International AAAI Conference on Web and Social Media, 8(1), 564–573. 10.1609/icwsm.v8i1.14507

[bibr73-17456916231180809] Zuckerman E. (2021). Why study media ecosystems? Information, Communication & Society, 24(10), 1–19. 10.1080/1369118X.2021.1942513

